# An open debate about the object and purpose of global health knowledge in the context of an interdisciplinary research partnership on HIV/STI prevention priorities in Peru

**DOI:** 10.1186/1744-8603-10-40

**Published:** 2014-05-21

**Authors:** Eliana Barrios Suarez, Carmen Logie, Jose F Arocha

**Affiliations:** 1 Lyle S. Hallman Faculty of Social Work, Wilfrid Laurier University, 120 Duke Street West, N2H 3W8 Kitchener, ON, Canada; 2 Factor-Inwentash Faculty of Social Work, University of Toronto; Adjunct Scientist, Women’s College Research Institute, Women’s College Hospital, University of Toronto, Toronto, ON, Canada; 3 School of Public Health and Health Systems, University of Waterloo, Waterloo, ON, Canada

**Keywords:** Interdisciplinary research, Global health knowledge, International collaborations, HIV prevention, Peru

## Abstract

**Background:**

With the failure of the latest vaccine trial, HVTN-505, HIV prevention efforts remain critical. Social and structural factors contributing to HIV and STI transmission include stigma regarding sexual violence, HIV infection and sexual orientation. For instance, HIV prevention and overall sexual health programs in Peru have been implemented yet key populations of youth (sex workers, male and transgender youth) continue to be overrepresented in new cases of HIV and STI. This suggests that interventions must take new directions and highlights the need for additional research.

**Discussion:**

While interdisciplinary, international research collaborations often are indicated as best practice in developing new knowledge in global health and an important component of the leadership in health systems, this does not mean they are free of challenges. In this debate we document our reflections on some of the challenges in developing an interdisciplinary and international research team to understand HIV and STI prevention priorities among youth in two culturally diverse cities in Peru: Lima, the capital city, and Ayacucho, in the Andean region.

**Summary:**

Rather than offering solutions we aim to contribute to the debate about the object and purpose of global health research in the context of developing international research partnerships that genuinely promote a reciprocal and bidirectional flow of knowledge between the Global South and the Global North, and researchers at intersections of these locations.

## Background

With the failure of the latest vaccine trial, HVTN-505, HIV prevention efforts remain critical [1]. While interdisciplinary, international research collaborations often are indicated as best practice in developing new knowledge in global health [2] and an important component of the leadership in health systems, this does not mean they are free of challenges. Rather than offering solutions we aim to contribute to the debate about the object and purpose of global health research [3,4] in the context of developing international research partnerships. Despite global efforts to prevent, treat, and ultimately cure HIV, the epidemic today affects 34 million people worldwide, including 1.9 million people living in Latin America and the Caribbean [5]. Social and structural factors contributing to HIV and STI transmission are in a large part comprised of stigma associated with sexual violence, persons living with HIV (PLHIV) and sexual and gender minorities (SGM). Undeniably there is ongoing marginalization of, and stigma directed toward, SGM worldwide that preclude human rights and full access to health services [6,7]. This situation is a public health and human rights problem in low and middle income (LMI) countries such as Peru—our case study [8-11]. It is also a persistent concern in high income countries (HIC) such as the US and Canada [12-15], demonstrating the transnational nature of these issues [16]. UNAIDS [17] reported that in 2011, of 74,000 PLHIV in Peru, 50,000 were men. The same report defined priority populations as including people involved in sex work, transgender persons, and men who have sex with men (MSM). It was estimated that most of the 5,800 new cases in 2011 belonged to these populations [17]. However, as in other global regions [18], statistics on gender-specific HIV incidence and prevalence are incomplete. Research has indeed indicated a ‘heterosexual bridging’ of HIV and syphilis transmission involving Peruvian MSM [19]. ‘Bridging’ in this case characterized the behaviors of men (bridgers) who reported sex with both men and women in the past year [19].

Although youth as a whole represent a global population at risk for HIV and STI infections, there is a great deal of variability within this developmental period and within high-risk subgroups [20]. In Peru, disaggregated statistics reveal increased risk for key populations of youth; male youth, sex workers, and other SGM populations are overrepresented in new HIV and STI cases in Peru [21]. The median age of persons living with AIDS in Peru in 2013 was 31 years old, which means that at least 50% were exposed to HIV before reaching 21 years of age [21]. These statistics point clearly to the importance of focusing on youth in HIV and STI prevention in Peru. Youth in Peru (15–29 years old) represent approximately 28% of the population of 8 million [22]. The United Nations Population Fund (UNPFA) released a report in 2012 that details the characteristics of the ‘demographic bonus’ of Peru. This concept refers to contexts where a majority of a population falls into the range of 15–59 years old and therefore is employable, the current context of Peru [22]. While the immediate consequence is an extremely competitive job market, the future is more concerning, as the current youth get older and the younger group occupies a much smaller proportion in the population pyramid. Youth in Peru are, however, a heterogeneous group: 48% are migrants living in large urban zones; only 1/3 are studying, while 83% work full or part-time; 37% of teenage girls that have only elementary education are also teen mothers; and 81% of youth in poverty are sexually active before 19 years of age [22]. While HIV and overall sexual health prevention programs targeting youth in Peru have been implemented [7], a review of local literature and discussions with local researchers and community groups indicated that the role of HIV stigma, sexual violence, cultural diversity and health literacy in the effectiveness of those programs warrants further attention. Of special concern is the dearth of policies that protect the sexual and reproductive health and rights of youth, in particular SGM youth [23]. Human rights are premised on the notion of human dignity; human dignity requires that individuals do not suffer from discriminatory legislation [24]. In Peru, the exclusion of youth in sexual health rights legal frameworks indicates that youth also lack overall civil and political rights, the core of human rights.

We are three social sciences and clinical researchers living and working in Canada; two of us are from South America (ES from Peru and JFA from Venezuela) and one from Canada (CL). While one of us has a program of research based in Peru (ES), the others are based in diverse global regions (e.g. Jamaica, South Africa, Haiti) and Canada. We represent different disciplines and universities, and travelled to Peru together to meet local agencies and researchers in May 2013. Our paper is based on reflections and dialogue that emerged during our research trip.

We plan to examine HIV and STI prevention priorities among youth in two culturally and geographically diverse cities in Peru: Lima, the capital city, and Ayacucho, in the Andean region. Peruvian researchers have reported on the intercultural diversity regarding how social and health indicators such as sexual violence, homophobia [25], religion, sexuality, sexual health and politics [26] are expressed and embraced in different provinces of Peru. The most significant differences were found between the coastal region (Lima) and the Andean region (Ayacucho), which is why we choose these two regions as the focus of our interest. This paper reflects our initial discussions on how best to prepare ourselves for international and interdisciplinary research in global health. After a preliminary trip exploring local priorities and local partnerships it was clear to us that in order to develop a viable cultural, age and gender appropriate HIV/STI prevention research agenda with key populations of youth in Peru, it is essential to work in partnership with community agencies, health researchers and knowledge users. Mindful of reported difficulties in facilitating youth participation—in particular vulnerable youth—in the creation of global health knowledge, which should be the foundation of international HIV policy [20,27], we intend to use interdisciplinary tools to facilitate this participation. Our discussion on how disciplinary understandings influence our practice as international health researchers is organized around the three thematic tensions on global health knowledge suggested by Rowson et al. [4]: What is the *object* of knowledge? What is the *purpose* of knowledge? What are the *types* of knowledge? The article is structured around our professional conversations and outlines the challenges we have experienced as health researchers in bridging the mentioned divides in global health in the context of our current project. Rather than offering solutions or recommendations it is a representation of our interpretations and deliberations; hence our exchanges are open for debate.

## Discussion

### Object of global health knowledge

The Global Fund for AIDS, Tuberculosis and Malaria (GFATM) is the keystone of HIV and STI prevention programs in LMI countries. Epidemiological analyses and cost-effectiveness evaluations of those interventions highlight MSM as the priority population in Peru [28]. However, research also pointed to other key populations at risk such as transgender women (transwomen) [29], female and male sex workers [30,21], pregnant women [31], female sexual partners of heterosexual/bisexual men [32,19], and street engaged youth [33]. Similarly to other countries [34-37] young MSM (YMSM) appear to have greater risks than MSM over 29 years old [20]. In addition, recent Peruvian statistics confirm regional variations of risk for all these populations [21] suggesting that interventions should also address region specific needs among these diverse populations. These considerations from our case study are somewhat in conflict with the emphasis in global health on commonalities, i.e. people’s health is affected by global forces and should be therefore approached using common strategies. While an emphasis on commonalities [as the object of global health] certainly facilitates the quick production of global health research and knowledge, we agree with Rowson and colleagues [4] that this may also pose risks regarding understanding of: a) the social context of health issues, b) the plurality of disciplinary perspectives on defining priority issues and c) the views of Southern populations on what health issues are significant.

In considering the social context of global health, the connection with human rights is central. While the complex policy and legal interpretations of the right to health [38] is beyond the scope of this discussion, it is important to note that Article 25 of the Universal Declaration of Human Rights [39], considers not only medical care but also access to housing, food, clothing, and social services as the right that everyone has for a standard of living adequate for health and well-being. However, the global health emphasis on medical interventions has been to some extent distant from a rights-based approach that emphasizes action to the pre-conditions of health and the underlying social determinants of health. This apparent dissonance has generated critiques on the universal validity of a global health framework [40]. For instance, global health knowledge in mental health has been questioned by psychiatrist Derek Summerfield because “it deflects attention from what millions of people worldwide might cite as the basis of their distress—for example, poverty and lack of rights” [41]. While some of these critiques have come from the medical field [42], others often arise from disciplinary fronts outside medicine [38], but all of these voices call for ‘new frontiers’ on global health politics [43]. These new frontiers are a reminder of Rowson et al.’s [4] call for a plurality of perspectives in global health knowledge. The authors of this paper indeed came from diverse disciplines, economics (ES), psychology (ES, JA), public health (JA), sociology (CL) and social work (ES, CL) and are combining their different epistemological frameworks to develop the current research project. For example, a social work strengths-based, anti-oppressive practice framework is congruent with a rights-based approach and emphasizes resilience as well as resistance [44]. This may seem inconsistent with the dominant framework of risk and vulnerability in health promotion, but combined they may safeguard the necessary inclusion of local participation in defining health problems and solutions and supporting the development of equitable partnerships where knowledge is co-created by knowledge users and researchers, in this case, by Peruvian youth and local and international researchers. We agree with Ouma and Dimaras [45] that to build equitable and mutually beneficial research relationships the views of local partners are highly important no matter which approach is taken. The inclusion of local youth, local researchers and local agencies as knowledge producers in our planned project reflects an attempt to transcend the typical North–south direction of global health knowledge and endorse instead a truly reciprocal, bi-directional flow. Our team itself, comprised of diasporic researchers from the global South living and working in the global North, also challenges the binary of global North vs. global South and highlights the intersectional and often complex, transnational identities of global health researchers.

### Purpose of global health knowledge

The purpose of global health research is contested. While some believe a researcher’s role is transactional and should benefit participants and avoid exploitation, others extend the scope of researchers engaged in global health to challenge conditions of injustice and oppression and to promote human rights [46,3,48]. Lavery et al. [3] propose a ‘relief of oppression’ framework that promotes freedoms, including social, economic and political opportunities. They argue that addressing injustice “is relevant to background conditions of injustice because it is precisely those conditions that so often give rise to the health and social circumstances that interest observational researchers working in global public health” [3]. This position implicates researchers from high income countries (HIC), or the global North, in addressing inequities that are at least in part shaped by inequitable international trade policies and laws and a history of colonization [3,48]. In contrast, Rothman et al. [15] argue that global health objectives should be ideologically neutral in order to avoid addressing politics. This approach points to the contextually varying definition of equity and norms regarding stratification across societies, suggesting that it is practically impossible to have a universal definition of equity [15]. While Standing et al. [49] reinforces the importance of grounding all discussions of human rights in local understandings of this concept, they also highlight that purposes of rights-based global health work include conceptual analyses, program and policy approaches, and advocacy for specific populations (e.g. people living with HIV).

Where HIV and STI are the foci, the majority of social and public health researchers concur that social and structural inequity shapes the vulnerability to infection among the most marginalized groups across all societies: SGM, youth, sex workers and prisoners [50,6,51]. This suggests that the ‘relief of oppression’ framework may be the most relevant for defining the purpose and guiding international HIV research projects [3]. For example, the ‘relief of oppression’ approach is particularly salient to addressing the HIV and STI prevention needs among SGM youth in Peru. With its focus on promoting freedoms, this framework has implications for research to promote a) political rights, including legal protection from discrimination and representation; b) social opportunities, such as access to social support; and c) access to economic opportunities [3]. These are all key to HIV prevention for SGM youth in Peru, and elsewhere [21,50,51]—and for health and human rights of SGM persons more generally [16].

There is a need for greater understanding about the significance and meaning of rights and equity in persons’ lives across diverse contexts [49]. Standing et al. [47] propose a research agenda that explores a) social conceptualizations of sexuality, rights and sexual and reproductive health (SRH), b) how groups, such as SGM persons, negotiate rights and wellbeing and c) how we can share lessons learned from diverse global contexts. In the Peruvian context this may translate into an exploration of socio-cultural norms regarding sexuality, rights, and HIV/STI in our 2 research areas—Lima and Ayacucho. It could also inform research that examines understandings of rights, stigma, resilience and HIV/STI among LGBT youth in Lima and Ayacucho, as well as initiatives for knowledge translation within and between countries. Kippax et al. [50] underscore the need to explore the intersection of socio-political and bio-medical perspectives on HIV prevention. From this angle the purpose of knowledge could be to examine multidisciplinary understandings of issues such as HIV prevention and socio-political mobilization [51]. These perspectives, while not exhaustive of the purpose and direction our proposed research could take, help to unpack our framework and approach to the role of researchers and provide a clear roadmap of the multi-directionality our research could take—from exploring the applicability of the ‘relief of oppression approach’, to exploring the meaning of rights and equity in Lima and Ayacucho, and/or applying a multi-disciplinary approach to informing HIV prevention.

### Types of global health knowledge: collaborative and participatory

HIV and STI prevention research crosses disciplinary and sectorial boundaries; such generation of knowledge of complex systems requires the participation of people with different academic backgrounds and interests, different methodologies and theoretical frameworks. Such diversity presents a challenge for the formation of health collaboratories—technology-supported research networks involving different stakeholders, such as researchers, healthcare providers, and community actors [52]. Although guidelines and experiences exist for ensuring the development of successful collaboratories [53,54] uniting all stakeholders in a general framework that takes into account each subspecialty constitutive of a collaboratory is a difficult task in and of itself. However, the use of health information technologies is an example of the diverse types of knowledge that can aid in the deployment and evaluation of such collaborative networks in global health projects by implementing innovative communication technologies and methodologies that promote coordination between health care providers/researchers and the target populations.

Consumer health informatics (CHI) is an emerging field of study that lies at the intersection between the social and behavioral sciences and information technology as applied to personal health issues [55]. However, unlike the most established areas of health informatics [56], which focus on hospital-based healthcare, CHI possesses several features that distinguish the field. First, its central focus is health information users, that is, patients and the lay public. Second, it cuts across the levels of individual and population health. Third, because of its focus on the layperson, one of its major concerns is with prevention of illness by fostering lifestyle changes and early screening behaviors. In this sense, CHI can serve to investigate and implement methods of empowering laypersons to become informed and skillful users of health information.

However, for populations that lack basic resources and are disempowered in society, such as street youth, SGM, and sex workers in LMI countries, adopting healthy lifestyle changes is particularly challenging. It may also reflect neoliberal ideologies that place the impetus for change on the individual vs. society. How can CHI help marginalized populations globally in becoming more informed and skillful users of health information and services? Apart from increasing accessibility through communication devices (e.g. cellular phones), CHI can also provide personalized information and reminders for health users, which they can use to reduce the distance between healthcare providers and themselves. CHI can also contribute to empowering patients by means of decision aids that help marginalized individuals to challenge the stereotypes that may be barriers to health care. For instance, CHI technologies can provide researchers and healthcare workers with ways of gathering specific patient information about users in a very secure and anonymous way, which allows the latter to share information without the threat of stigmatization. Furthermore, relevant to our project at hand is the use of peer-to-peer (P2P) technologies. These are based on utilizing a network of users for storing and distributing information, rather than a central repository or database; this can inform the infrastructure to develop and maintain social networks of people with similar lifestyles, interests, and social identities including sexual orientation and gender identity. In this regard, one potential implementation could be the development of secure user portals for SGM youth in Peru who can access such websites for accessing personal health information or as venues for interacting with healthcare providers and community activists.

However, although the CHI field has been heralded as an important contributor to the solution of many of the problems facing healthcare and medicine today, including HIV and STI prevention, CHI interventions have to meet several challenges when applied to complex “real life” situations, from access to health services [57] to the use of screening aids for basic health concerns [58]. One of the major challenges is the variability in the populations that are the target of CHI interventions in global health. For instance, in the context of our project in Peru, information has to be not only timely, but also tailored to the characteristics of SGM youth, especially those groups who need such interventions the most. It is encouraging that previous research in Peru indicated favorable attitudes among HIV positive groups about using the Internet, cell phones and Personal Digital Assistants for HIV health promotion interventions, pointing in particular to cell phones as feasible and culturally appropriate tools in resource-poor settings [30]. In short, problems regarding literacy, accessibility, usability of the electronic tools, and comprehension of the information provided by health care professionals are some of the issues to which CHI could contribute real solutions by devising different portals for SGM youth depending on their social environment (e.g., urban–rural), age (adolescents vs. adults), and other factors. For instance, studies have shown that by using methods of analysis of health information it is possible to tailor such information to specific populations to increase readability and comprehensibility [59]. For these strategies to be successful information should be adapted to the particular characteristics of the youth who are the target of the research and interventions, such as the appropriate literacy levels and degrees of comprehension, while matching the information usability to their learning styles. An example of this is provided by studies that improve people appropriate literacy levels and manipulate the cognitive load [60] necessary for ease of processing, such as improvements in coherence of information, propositional density, and use of illustrations [61]. In turn, by including users as generators of knowledge, we can start developing more systematic and potentially more sustainable research endeavors. CHI is merely one example of the bridging of social sciences and biomedical perspectives and the benefits that this interdisciplinary confluence can offer to HIV research [48] and ultimately to the study of globalization and health. The tensions of bridging individual focused interventions with social and structural level interventions for HIV and STI prevention with key populations has been articulated but is important to reiterate as there is increasing attention to biomedical and new prevention technologies [62-64].

## Summary

In contrast with the popular trend of focusing on the technical tools of “how to do” global health research this debate focuses instead on thoughts about the “why” and “what” of global health knowledge which are essential deliberations for HIV research and prevention. Balancing the various priorities and strengths of social and biomedical sciences, including the multiple levels of focus (individual, social structural) and multiple actors involved (youth, parents, health care providers, community-based organizations) are all significant factors to be considered when situating global health discussions. By discussing the tensions surrounding what the object and purpose of global health knowledge is, we highlight the connection of global health with an international human rights framework and with interdisciplinary and cross-sectoral debates. By emphasizing a context-specific, participatory, and interdisciplinary approach in global health we are also considering that global health problems have distinctive significance and solutions in different international settings. We contend that where knowledge is co-created by knowledge users and researchers a multidirectional flow of global health knowledge can be achieved. With globalization we have witnessed an increased global flow and exchange of services and practices generally from North to South; we hope to encourage a more humbling approach in global health research, that is, a reciprocal, multidirectional flow of knowledge from the South to the North.

## Competing interests

Authors declare no competing interests.

## Authors’ contributions

ES and CL formulated the original idea for this debate article. ES wrote the first draft and CL and JFA contributed with additional sections. ES prepared the final draft and all authors read and approved the final manuscript.
